# Obstacle Avoidance amongst Parkinson Disease Patients Is Challenged in a Threatening Context

**DOI:** 10.1155/2013/787861

**Published:** 2013-05-15

**Authors:** Jon B. Doan, Natalie de Bruin, Sergio M. Pellis, Oksana Suchowersky, Ian Q. Whishaw, Lesley A. Brown

**Affiliations:** ^1^Engineering and Human Performance Laboratory, Department of Kinesiology and Physical Education, University of Lethbridge, Lethbridge, AB, Canada T1K 3M4; ^2^Canadian Centre for Behavioural Neuroscience, University of Lethbridge, Lethbridge, AB, Canada T1K 3M4; ^3^Hotchkiss Brain Institute, University of Calgary, Calgary, AB, Canada T2N 1N4; ^4^Departments of Medicine (Neurology) and Medical Genetics, Faculty of Medicine and Dentistry, University of Alberta, Edmonton, AB, Canada T6G 2R3

## Abstract

We examined whether people with Parkinson disease (PD) have difficulty negotiating a gait obstruction in threatening (gait path and obstacle raised above floor) and nonthreatening (gait path and obstacle at floor level) contexts. Ten PD patients were tested in both Meds OFF and Meds ON states, along with 10 age-matched controls. Participants completed 18 gait trials, walking 4.7 m at a self-selected speed while attempting to cross an obstacle 0.15 m in height placed near the centre point of the walkway. Kinematic and kinetic parameters were measured, and obstacle contact errors were tallied. Results indicated that PD patients made more obstacle contacts than control participants in the threatening context. Successful crossings by PD patients in the threatening condition also exhibited kinematic differences, with Meds OFF PD patients making shorter crossing steps, with decreased initiation and crossing velocities. The findings from this study lend support to the theory that PD patients rely on directed attention to initiate and control movement, while providing indication that the motor improvements provided by current PD pharmacotherapy may be limited by contextual interference. These movement patterns may be placing PD patients at risk of obstacle contact and falling.

## 1. Introduction

Epidemiological investigation indicates that Parkinson disease (PD) patients experience more falls than either age-matched healthy controls or individuals with other neuropathologies, including spinal disorders, epilepsy, multiple sclerosis, stroke, and motor neuron disease [1]. For patients with PD, fall occurrences and increased fear of falling are frequent in situations with complex or threatening context [2], with contact with an obstacle presenting a major cause of falls among PD [1, 3]. Task demands, such as the inherent characteristics of the obstacle to be crossed as well as constraints imposed by the general environment surrounding the obstacle and task, contribute to context [4] and exacerbate motor disturbances amongst PD patients [5]. Previous studies have shown that neurotypical adults adopt conservative strategies for standing [6, 7], walking [8], and obstacle crossing [9] when behaving in a context that threatens increased physical consequences as a result of a fall. In contrast, PD patients have exhibited increased postural instability [10] and gait disturbance [11] when concurrently challenged with a cognitive or motor demand. It is probable that threatening context may exacerbate any obstacle negotiation deficits that exist for PD patients. While PD pharmacotherapy reduces classical parkinsonian symptoms [12], some functional movement parameters remain insensitive to dopamine replacement [13, 14]. Furthermore, improvements enabled by PD medication can be compromised by challenging context [15, 16]. This compromise can lead to instability during standing and walking in activities of daily living, increasing fall risk. This phenomenon has been documented in previous work [17].

The purpose of this study was to investigate changes in obstacle crossing behaviour amongst the meds ON and meds OFF PD patients in response to task context. We had patients step over a walking-surface obstacle in two contexts: at floor level and on a raised walking platform, previously identified as sufficient to threaten participants' sensorimotor system, and elicit changes in motor strategy [6, 9]. We hypothesized that threatening context would have stronger influence on obstacle crossing than dopamine replacement, resulting in obstacle negotiation deficits amongst both meds ON and meds OFF PD patients.

## 2. Methods

### 2.1. Participants

Ten participants with idiopathic PD (PD; age: 69.7 ± 10.3 years) and ten age-matched controls (CTRL; age: 68.8 ± 8.4 years) served as subjects. All participants were informed on the nature of the study and provided written consent. The Human Research Ethics Committee of the University of Lethbridge had previously approved all procedures.

All PD patients were receiving dopaminergic and associated medication as PD management (Table 1), and each PD subject was tested meds OFF (>12 h removed from last dose) and meds ON (between 1 h and 2 h following regular dose) in the same laboratory visit (same day). All patients were tested in the OFF then ON order for patient's practicality and comfort. Quality of ON condition was confirmed by patient's self-report and clinical assessment. The Unified Parkinson Disease Rating Scale motor scores (UPDRS-III) assessed at time of testing are provided in Table 1.

#### 2.1.1. Apparatus

**Figure 1:**
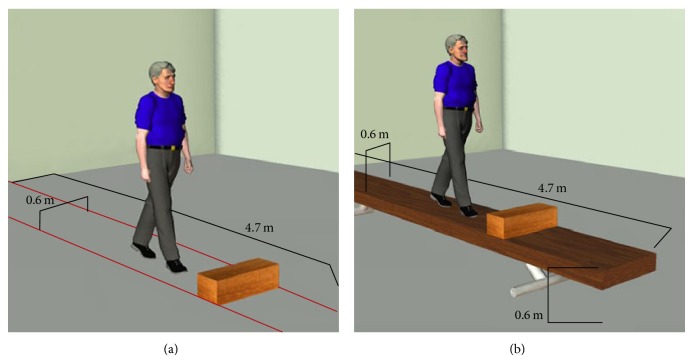
Conditions of environmental context. (a) low postural threat and (b) high postural threat. Subjects wore a full-body safety harness in all trials. In high threat trials, the harness was attached to a rolling coupling on an overhead track (not shown).

Participants started in a standing posture at the beginning of a 4.7 m long, 0.6 m wide walkway, with each foot positioned such that the lateral malleolus was aligned with the centre line of a separate force plate (Kistler Products). Threatening context was imposed by increasing the potential negative result of a fall, as empirically established in previous human movement studies [6–9]. In the high condition, the test walkway was solidly supported 0.7 m above the ground, and the force plates were raised to an equal height on a hydraulic lift. In the low condition, the walkway was outlined on the laboratory floor with continuous tape borders (Figure 1). A ramp (0.9 m length, 5° angle of declination) was positioned at the start of the walkway, flush with the anterior edge of the lowered force plates, to allow for gradual vertical displacement from low force platform height (0.09 m) to low walkway height (0.00 m). The obstacle was a rigid foam block (0.15 m high, 0.60 m wide (perpendicular to gait path), and 0.15 m long), approximately equal in height and length to a North American concrete parking curb.

All participants wore a safety harness for all trials, and that harness was tethered to an overhead rolling coupling to prevent falls to the ground. Participants also wore vision-occluding goggles (PLATO, Translucent Technologies, Toronto, ON) that initially concealed the presence or absence of the gait obstacle, to control for the preplanning of obstacle negotiation strategy. During practice trials, participants were familiarised with the preparatory stimulus (opening of the goggles) and the imperative stimulus (audio signal). In experimental trials, the goggles were initially set to closed. Once the investigator had positioned the obstacle (for obstructed trials) or feigned placing the obstacle (nonobstructed trials), a second experimental investigator informed the participant that a new trial was set to begin. At a random interval following this instruction, the goggles were opened. The imperative stimulus sounded 0 ms, 500 ms, or 1000 ms after goggles opening, with all subjects receiving the same number of trials at each latency (*n* = 3) in the same random order.

#### 2.1.2. Procedure

 Subjects walked at a self-selected speed along the walkway in each of the high and low conditions, performing a block of 18 trials in each condition (36 trials total). Order of threat condition was counterbalanced between subjects. Obstacle trials were further randomized in each threat condition, such that 9 of 18 trials in each threat condition involved obstacle negotiation and nine were nonobstructed trials. All subjects performed two practice trials prior to the start of each threat condition. Obstacle position was chosen at a point on the walkway equal to or greater than three stride lengths from the point of gait initiation for each subject, as determined during practice trials. This positioning allowed participants to transition from gait initiation to a stable gait pattern and provided adequate time for obstacle negotiation behaviour to reach a stable level [18]. A fixed posture with arms loosely crossed in front of the body was used to limit obstruction of markers.

#### 2.1.3. Data Collection

 Participants were outfitted with passive infrared-reflective markers at the following anatomical locations: bilaterally at the anterior end of the shoe, the lateral malleolus, the posterior end of the shoe, the lateral epicondyle of the femur, the greater trochanter, the ulnar styloid, the lateral epicondyle of the humerus, and the acromion process and unilaterally at the sternal notch and the forehead. A single marker was also placed in the top center of one sagittal face of the obstacle. Positional data were collected using a 6-camera infrared motion analysis data collection system (Peak Motus 2000, Peak Performance Technologies, Englewood, CO), with a collection frequency of 120 Hz. Synchronized digital video recordings of each trial were made in the sagittal and frontal planes for qualitative scoring of obstacle negotiation. Kinetic data for gait initiation were also captured from the force plates at a collection frequency of 600 Hz, in synchrony with an analog signal split from the audio imperative stimulus. 

 Behavioural coding of obstacle contact was completed from video by three individual judges and corroborated with kinematic analysis of the obstacle marker displacement. Trials where a participant contacted the obstacle were removed from further kinematic analysis as were any trials that could not be successfully postdigitized. Given these reductions, the total number of trials included in kinematic analyses was PD OFF—74, 69; PD ON—76, 65; CTRL—79, 75 for low and high conditions, respectively.

**Figure 2:**
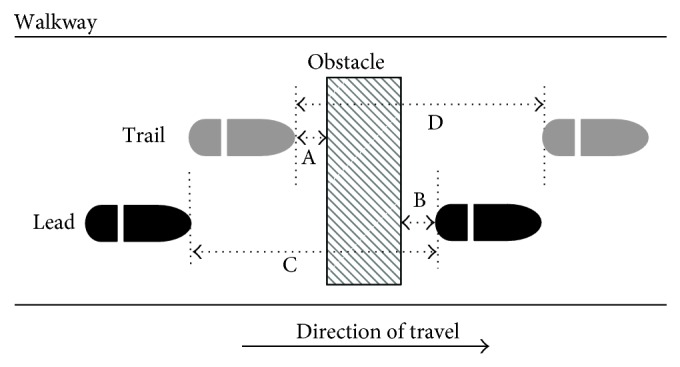
Top-down view illustration of obstacle crossing, with horizontal spatial measures of interest. Obstacle is marked in diagonal lines, while lead and trail feet are indicated by black and gray ovals, respectively. Measures shown are (A) trail foot precrossing clearance (*D*_PRE_), (B) lead foot postcrossing clearance (*D*_POST_), (C) lead foot crossing step length (CR_LEAD_), and (D) trail foot crossing step length (CR_TRAIL_). Step lengths were averaged (SL). Not shown is horizontal centre of mass crossing velocity (CV_COM_), and vertical toe clearance (*D*_LEADVERT_).

 Kinetic and kinematic data were processed using custom algorithms (MATLAB, The Mathworks, Natick, MA, USA). Raw displacement data were visually inspected and interpolated as required then filtered using a fourth-order Butterworth low pass digital filter with a cutoff frequency of 10 Hz. Velocity data were calculated through the differentiation by finite differences. Pertinent kinematic measures assessing obstacle approach and obstacle negotiation in both the lead limb (first limb across obstacle) and the trail limb (second limb across obstacle) are illustrated in Figure 2. They include the precrossing measure of horizontal distance from rear edge of obstacle to trail toe off (*D*_PRE_), the crossing measure of vertical distance between top of obstacle and lead toe (*D*_VERT_), and the postcrossing measure of horizontal distance from front edge of obstacle to lead heel strike (*D*_POST_), along with determinations of crossing step length (SL) from trail toe off to lead heel strike and horizontal velocity (CV_COM_) of whole body centre of mass at crossing. Centre of mass was determined using participant mass and segment mass proportions from Winter [19]. Gait initiation rate was expressed as a time (unload time), being the difference in time between the imperative stimulus signal and a zero vertical force reading from one of the force plate pair.

#### 2.1.4. Statistical Analysis

 Separate *χ* ^2^ analyses were used to examine group and threat effects in the obstacle contact frequency counts. A mixed model MANOVA comparison was conducted on the kinematic measures, with the followup between group (PD OFF versus CTRL; PD ON versus CTRL) × threat (low versus high) univariate ANOVAs and within group (PD OFF, and PD ON) × threat (low versus high) repeated measure ANOVAs performed, with a corrected level of significance of *α* = .017 for multiple comparisons. 

## 3. Results

### 3.1. Unobstructed Trials

**Figure 3:**
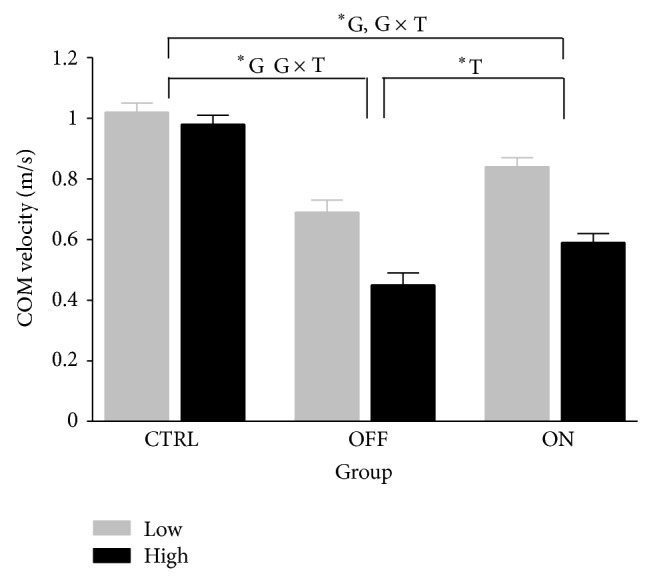
Horizontal centre of mass velocity for two strides prior to obstacle crossing stride for neurologically normal older adults (CTRL; *n* = 10), the MEDS off Parkinson disease patients (OFF; *n* = 10), and the same patients with normal medication levels restored (ON; *n* = 10) in unobstructed walking trials. G and T indicate significant main effects (*P* < .017) for group and threat, while G × T indicates a significant interaction (*P* < .017) of group and threat effects.

Unobstructed walking trials in low and high threat conditions were considered as a baseline in the current study. Group mean values for horizontal velocity at the centre of mass are shown in Figure 3. 

#### 3.1.1. PD OFF versus CTRL

PD OFF subjects had a slower COM horizontal velocity than CTRL subjects (F(1, 18) = 80.76, *P* < .001; CTRL = 1.01 m/s; PD  OFF = 0.58 m/s). Univariate follow-up tests revealed that these measures were supported by group × threat interactions ((F(1, 18) = 4.90, *P* < .05). PD OFF walked significantly slower in the high condition.

#### 3.1.2. PD ON versus CTRL

PD ON walked slower (F(1, 18) = 25.75, *P* = .00; CTRL = 1.01 m/s; PD  ON = 0.72 m/s) than CTRL subjects. A group × threat interaction indicated that the manipulation of postural threat affected gait velocity amongst PD ON subjects differently than CTRL subjects (F(1, 18) = 5.25, *P* < .05). PD ON demonstrated significantly slower walking speed in the high condition.

#### 3.1.3. PD ON versus PD OFF

A significant main effect for threat on COM velocity (F(1, 18) = 12.11, *P* < .05) was revealed through the multivariate analysis. Group and group × threat effects did not exist (F(1, 18) = 2.48, *P* > .05 and F(1, 18) = .92, *P* > .05, resp.).

### 3.2. Obstructed Trials—Approach

**Figure 4:**
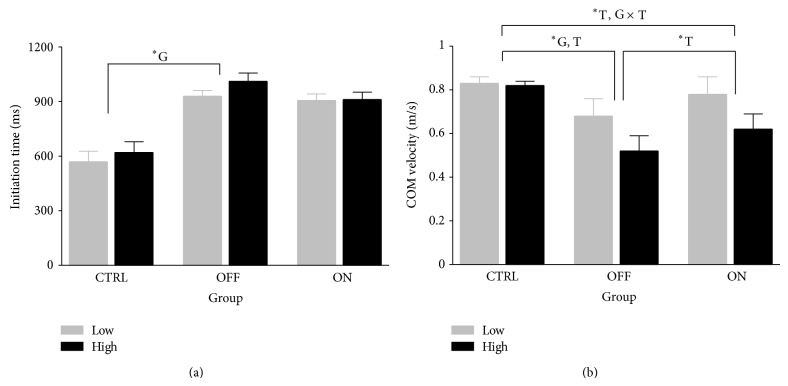
(a) Gait initiation time for neurologically normal older adults (CTRL; *n* = 10), the MEDS off Parkinson disease patients (OFF; *n* = 10), and the same patients with normal medication levels restored (ON; *n* = 10). Unload time is the elapsed time between the imperative “go” stimulus and a zero reading for vertical force from one of the force plate pair. (b) Horizontal centre of mass velocity for two strides prior to obstacle crossing stride for neurologically normal older adults (CTRL; *n* = 10), the MEDS off Parkinson disease patients (OFF; *n* = 10), and the same patients with normal medication levels restored (ON; *n* = 10). G and T indicate significant main effects (*P* < .017) for group and threat, while G × T indicates a significant interaction (*P* < .017) of group and threat effects.

There were no group or threat-based differences for gait initiation rate during obstructed trails (Figure 4(a)). PD OFF did produce significantly lower COM velocities during obstacle approach compared to CTRL (F(1, 18) = 11.350, *P* = 0.003). All three groups decreased COM approach velocity in the high condition (Figure 4(b); PD ON/CTRL: F(1, 18) = 15.632, *P* = .001; PD OFF/PD ON: F(1, 18) = 17.944, *P* = .002). Larger decreases in COM approach velocity amongst PD patients in the high condition led to a threat × group interaction in the PD ON/CTRL comparison (F(1, 18) = 11.408, *P* = .003). 

### 3.3. Obstructed Trials—Crossing

#### 3.3.1. Obstacle Contact Errors

**Figure 5:**
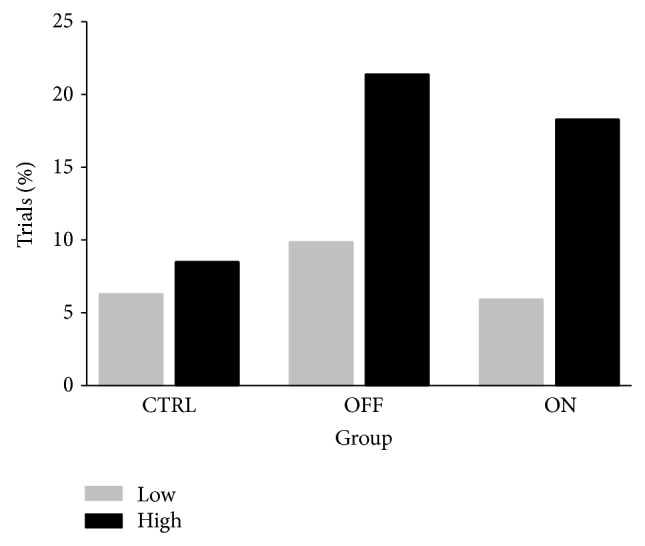
Obstacle negotiation error rates for neurologically normal older adults (CTRL; *n* = 10), the MEDS off Parkinson disease patients (OFF; *n* = 10), and the same patients with normal medication levels restored (ON; *n* = 10). In both the low and high environmental threat conditions, the obstacle dimensions were identical.

 PD OFF had a high frequency of obstacle contacts in the high condition; in total, 21.3% of trials compared to 9.9% observed in low (*χ* ^2^(1) = 4.05, *P* < .05). PD ON also made more frequent obstacle contact in high (observed in 18.3% of trials) than in low (5.9% of trials) (*χ* ^2^(1) = 5.49, *P* < .05). Conversely, CTRL had few obstacle contacts in both the high (8.5% observed) and low (6.3% observed) conditions, and these differences did not reach significance (*χ* ^2^(1) = 0.32, *P* > .05). Obstacle contact frequencies are presented in Figure 5.

#### 3.3.2. Kinematic Analysis

Kinematic parameters for low and high condition obstacle crossing are presented in Table 2.

#### 3.3.3. PD OFF versus CTRL

 PD OFF was significantly slowed in obstacle crossing velocity compared to CTRL (F(1, 18) = 11.317, *P* = .003), regardless of threat condition. Both PD OFF and CTRL reduced CV_COM_ (F(1, 18) = 14.481, *P* = .001) while negotiating the obstacle in the high condition. Compared to CTRL participants, PD OFF used a smaller precrossing margin (*D*_PRE_; F(1, 18) = 10.941, *P* = .004) with a smaller crossing step (SL; F(1, 18) = 10.993, *P* = .004) in both conditions. PD OFF and CTRL both tended to reduce *D*_PRE_ in the high condition (F(1, 18) = 3.897, *P* = .064). In contrast, CTRL increased postobstacle horizontal clearance of the lead heel in the high condition (*D*_POST_; 33 ± 8 cm, as compared to 23 ± 5 cm in low), where PD OFF produced horizontal heel clearance values of similar small magnitudes in either condition (15 ± 2 cm in low, 14 ± 2 cm in high). Both groups slightly decreased vertical obstacle clearance in the high condition. 

#### 3.3.4. PD ON versus CTRL

 PD ON and CTRL both decreased the crossing velocity in the high threat condition (F(1, 18) = 25.988, *P* < .001). PD ON used smaller crossing steps than CTRL (SL; F(1, 18) = 45.247, *P* < .001), but both groups decreased crossing step length in the high condition (F(1, 18) = 12.671, *P* = .002). In contrast, PD ON used a smaller preobstacle margin than CTRL in both threat conditions (*D*_PRE_; F(1, 18) = 9.510, *P* = .006). Postobstacle lead heel horizontal clearance approached a group  ×  threat interaction (F(1, 18) = 5.130, *P* = .036), with PD ON leaving smaller lead heel clearance in the high condition (11 ± 2 cm, compared to 16 ± 2 cm in low), while CTRL increased lead heel clearance in high obstacle crossing (33 ± 8 cm, compared to 23 ± 5 cm in low).

#### 3.3.5. PD OFF versus PD ON

 PD OFF and PD ON used significantly slower whole body COM obstacle crossing velocity (CV_COM_; F(1, 9) = 10.252, *P* = .010) in the high condition. PD patients also used a smaller crossing step in the high condition (SL; F(1, 9) = 17.663, *P* = .002), with PD ON using smaller crossing steps than PD OFF in both conditions (F(1, 9) = 30.111, *P* < .001). Both groups exhibited non-significant decreases in precrossing toe clearance, vertical clearance, and postcrossing heel clearance in the high condition.

## 4. Discussion

The results of this study agreed with our hypotheses, indicating that threatening context challenged locomotion amongst people living with the Parkinson disease and that obstacle crossing errors were increased, while obstacle crossing kinematics, specifically obstacle clearance distances and velocity, was decreased during threatened context trials. In addition, motor improvements potentiated amongst PD patients through conventional pharmacotherapy were not uniformly maintained in the threatening context. PD ON used small preobstacle clearance margins and small crossing steps to negotiate the obstacle. We suggest that motor improvements among medicated PD patients can be compromised by context. Previous studies have established that PD motor deficits are manifest in multiple aspects of gait, including initiation [20], steady state [21], and termination [22]. We suggest that the changes in obstacle avoidance behaviour observed among PD patients in the threatening context may be the result of constraints induced when some attention is directed toward a threatening environment [12]. Previous studies have used dual task paradigms to elicit similar obstacle negotiation deficits among neurotypical populations [23, 24]. 

The main finding of this study is that threatening context appears to be detrimental for PD patients. In healthy adults, perception and classification of threat require attentional resources, with higher threat requiring greater resources [25]. For PD patients, the diversion of attentional resources to threatening context may lead to an attentional resource conflict, as previous studies have suggested that patients have adapted to use directed attention to initiate and control movements [10, 11, 26]. Subdividing attention may exceed available capacity, especially amongst moderate to severe PD patients, who have been shown to have decreased executive function [27]. 

It is possible that the increased errors in the high condition are the result of arousal and anxiety induced by threatening context. Increased anxiety may also be a partial product of the safety precautions that surround the high condition, namely, the need for the overhead tether. Previous studies from our laboratory [7–9] and others [6] have shown that anxiety-provoking contexts can lead to kinematic changes in behaviour. One limitation of the current study is the lack of state or trait anxiety measures, including fear of falling, amongst participant groups. Previous research has shown that the PD patients exhibit higher levels of anxiety [28] and a heightened fear of falling in threatening contexts [29]. While it is possible that the errors observed amongst PD patients completing threatened trials in this study are a partial result of raised anxiety, we did not observe changes in success rates between the low and high conditions for healthy normal adults. This finding contradicts previous research and suggests that the threat manipulation imposed in this study was not sufficient to invoke performance-inhibiting anxiety amongst the non-Parkinson participants. It is possible that both attentional interference and increased anxiety contribute to the deficits observed amongst PD patients in the threatening context and that some portion of the diverted attention is consumed by perception and interpretation of threatening context. 

 Our results show that current pharmacological treatment of PD allowed patients to achieve fewer obstacle contact errors and improve gait kinematics, though these improvements failed to reach levels equal to control participants. Furthermore, threatening context appeared to have the capacity to limit medication benefits, reducing obstacle crossing success rates and crossing kinematics for MEDS on PD patients to similar levels as MEDS off PD patients. Previous work has indicated that temporal aspects of gait (e.g., stride cadence and stride event durations) are less sensitive to dopamine replacement [13, 30]. Given the critical importance of gait cadence and response timing in obstacle negotiation [18], it follows that this activity may still be deficit for MEDS on PD patients if cadence and timing are only moderately improved with medication. One limitation of the current study is incomplete information on levodopa dosage levels, eliminating the possibility to fully consider dose-response relationships or possible confounders for persistent MEDS on deficits. Despite this limitation, it is possible that the increased deficits observed for medicated PD in the threatening environment reflect a situational dysfunction in the nondopaminergic neural processes at work in this environmental context. We believe that executive attentional resources are the nondopaminergic assets that are being overloaded by concurrent attentional demands from perceived environmental threat and directed focus on task control. 

## 5. Conclusion

Our findings show that obstacle negotiation amongst PD patients is compromised in a threatening context. PD patients exhibited more obstacle contacts, decreased obstacle crossing clearance margins, and decreased approach and crossing velocities when walking in a threatening condition. Conventional PD pharmacotherapy failed to reduce obstacle contacts or increase obstacle clearance in the threatening context. Interference resulting from the attention diverted to threatening context plus the directed attention used by PD patients to initiate and control movement may be the cause of obstacle negotiation deficits.

## Figures and Tables

**Table 1:** Clinical information of the Parkinson disease patient group.

Patient	Age (yr)	Disease duration	Sex	UPDRS-III∗		Symptoms (OFF)			Medication
				ON	OFF	Bradykinesia	Action Tremor	Resting Tremor	
1	80	15	M	28	45	Y	Y	Y	Levodopa Levodopa (sustained release)
2	69	4	M	18	40	Y	Y	Y	Levodopa
3	76	8	M	6	24	Y	Y	N	Levodopa Levodopa (sustained release) Pramipexole
4	75	1	M	6	17	Y	N	Y	Levodopa
5	81	7	M	16	33	Y	Y	Y	Levodopa Pramipexole
6	54	10	F	5	14	Y	N	Y	Levodopa Pergolide mesylate Amantadine
7	54	22	F	21	43	Y	Y	Y	Levodopa Pramipexole
8	80	2	F	38	54	Y	Y	Y	Levodopa Amantadine
9	63	2	F	22	58	Y	Y	Y	Levodopa
10	65	11	F	21	34	Y	Y	N	Levodopa Pramipexole
Mean (SD)	69.7 (10.3)	8.2 (6.6)		18.1 (10.5)	36.2 (14.7)				

^*^The Unified Parkinson Disease Rating Scale-III (motor component—questions 18–31), with higher scores indicative of greater motor deficit.

**Table 2:** Summary of kinematics (mean [SEM]) for obstacle negotiation.

Measure	CTRL		PD OFF		PD ON		G	T
	Low	High	Low	High	Low	High		
*D*_PRE_ (m)	0.57 [0.05]	0.44 [0.06]	0.36 [0.05]	0.32 [0.04]	0.35 [0.07]	0.27 [0.06]	A, B	
*D*_VERT_ (m)	0.21 [0.05]	0.19 [0.01]	0.19 [0.05]	0.15 [0.02]	0.17 [0.03]	0.16 [0.01]		
*D*_POST_ (m)	0.23 [0.05]	0.33 [0.08]	0.15 [0.02]	0.14 [0.02]	0.16 [0.02]	0.11 [0.02]		
SL (m)	0.87 [0.06]	0.81 [0.04]	0.69 [0.02]	0.66 [0.04]	0.57 [0.03]	0.40 [0.03]	A, B, C	B, C
CV_COM _(m/s)	0.68 [0.05]	0.54 [0.03]	0.49 [0.04]	0.39 [0.03]	0.52 [0.06]	0.40 [0.05]	A	A, B, C

Reported measures are: *D*_PRE_: horizontal distance from rear edge of obstacle to trail toe off, *D*_VERT_: vertical distance between top of obstacle and lead toe, *D*_POST_: horizontal distance from front edge of obstacle to lead heel strike, SL: crossing step length from trail toe off to lead heel strike, and CV_COM_: horizontal velocity of whole body centre of mass at crossing. Results of group comparisons are provided in column G and results of threat comparisons in column T. Statistical comparisons indicating a significant difference in results between the PD OFF and CTRL groups are marked with an A, comparisons between PD ON and CTRL with a B, and repeated measures comparisons within PD OFF and PD ON with a C.

A: CTRL/OFF, *P* < .017.

B: CTRL/ON, *P* < .017.

C: OFF/ON, *P* < .017.
